# SARS-CoV-2-Induced Autoimmune Hepatitis

**DOI:** 10.7759/cureus.38932

**Published:** 2023-05-12

**Authors:** Nora Martini, Pranav Singla, Elizabeth Arbuckle, Geetika Goyal, Qiang Liu, Maria L Santos-Zabala, Hanady Zainah

**Affiliations:** 1 Internal Medicine, St. John's Riverside Hospital, Yonkers , USA; 2 Internal Medicine, St. John's Riverside Hospital, Yonkers, USA; 3 Internal Medicine, Lake Erie College of Osteopathic Medicine, Greensburg, USA; 4 Gastrointestinal and Liver Pathology, Montefiore Medical Center, Albert Einstein College of Medicine, New York City, USA; 5 Surgical Pathology, University of Pennsylvania Perelman School of Medicine, Philadelphia, USA; 6 Pathology and Laboratory Medicine, Montefiore Medical Center, Bronx, USA; 7 Pathology, St. John's Riverside Hospital, Yonkers, USA

**Keywords:** serology testing, periportal fibrosis, gastrointestinal and liver pathology, covid-19 and jaundice, covid, autoimmune hepatitis

## Abstract

Few case reports discuss the incidences of autoimmune hepatitis (AIH) in patients after SARS-CoV-2 infection. Here, we present a case of SARS-CoV-2-induced AIH in a male patient who came into the emergency department with complaints of weight loss, poor oral intake, nausea, dark-colored urine, clay-colored stools, and scleral icterus, which began two weeks after he tested positive for SARS-CoV-2 PCR. Liver biopsy and subsequent histology confirmed the diagnosis of AIH with the most probable etiology being SARS-CoV-2 infection. The patient was treated with N-acetylcysteine (NAC) and steroids with clinical improvement and eventual discharge home. Our goal is to provide a clinical presentation, treatment, and outcome in a patient with SARS-CoV-2-induced AIH.

## Introduction

A recent (April 2022) literature review found 32 cases documented of autoimmune hepatitis (AIH) after SARS-CoV-2 vaccination, [1] and a handful more cases have been published since. However, only a couple of cases have been described detailing SARS-CoV-2 infection causing AIH [2]. AIH is not a new phenomenon, but SARS-CoV-2-related disease is. AIH, like most autoimmune diseases, has a female predominance, yet estimates of the incidence and prevalence of AIH in the United States are scattered [3]. The pathogenesis of a disease is poorly understood but believed to be a result of environmental, viral, or chemical triggers (in this case SARS-CoV-2 infection) in a genetically susceptible individual [4].

Diagnosis requires clinical presentation, at least one elevated transaminase greater than two times the upper limit of normal, and a minimum of one positive serological marker or an increased total IgG or gamma-globulin levels. Confirmation is done with a liver biopsy. Histologic findings of AIH are nonspecific but may include portal mononuclear cell infiltrate, periportal lesions, bile duct changes, plasma cell infiltrates, and fibrosis [5].

Objective

We seek to portray a unique case of AIH in a patient who recently had a SARS-CoV-2 infection and describe the clinical and histological findings associated with this condition.

## Case presentation

A 42-year-old male with a past medical history of hypertension presented to the emergency department with a complaint of fatigue, weight loss, poor oral intake, nausea, dark-colored urine, clay-colored stools, and scleral icterus, for three-week duration. His symptoms started two weeks after asymptomatic routine employment PCR testing positive for SARS-CoV-2. These symptoms were accompanied by darkening of his skin, fatigue, nausea, and vomiting. He also endorsed an approximate 20 lb weight loss which he attributed to a feeling of fullness despite poor oral intake.

The patient was vaccinated for hepatitis B, hepatitis A, and SARS-CoV-2 (two doses of AstraZeneca over one year prior to presentation). He did not have a history of non-alcoholic steatohepatitis, alcohol use, or illicit drug use disorders. He had no history of recent travel. His only medication is perindopril/indapamide. 

Physical exam was notable for scleral icterus, enlarged nontender submandibular lymph nodes bilaterally, prominent hepatosplenomegaly, and jaundice. The skin exam did not demonstrate any rashes. He was alert and oriented to person, place, time, and situation, and had no mental status changes. He was afebrile at 98.1F, heart rate of 84 beats per minute, blood pressure of 128/89, respiratory rate of 18, and oxygen saturation of 99% on room air. 

**Figure 1:**
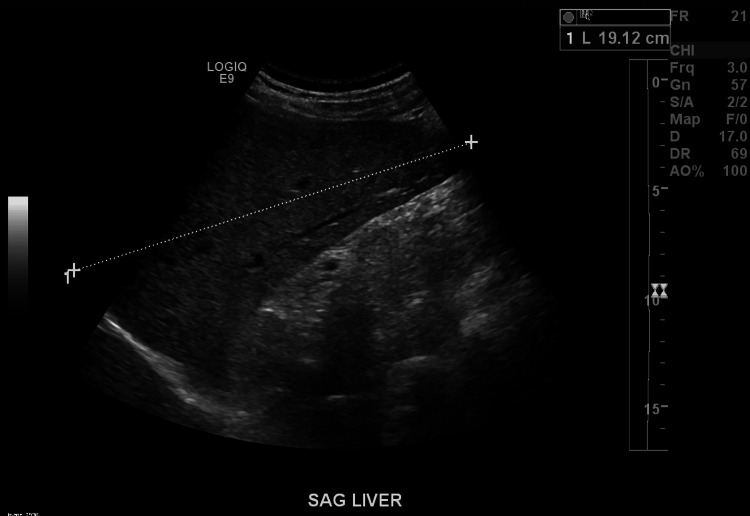
Abdominal ultrasound

Laboratory testing demonstrated elevated transaminases, markedly elevated bilirubin level, elevated antinuclear antibodies (ANA), elevated total IgG level, and elevated anti-smooth muscle antibodies. Hepatitis serology indicated no active infection with hepatitis A, B, or C. SARS-CoV-2 PCR was negative. Cytomegalovirus (CMV) PCR in the blood was positive; however, CMV immunohistochemical staining on biopsy was negative. The ferritin level was elevated (Table 1). Abdominal ultrasound showed a liver with a homogeneous echotexture and enlargement measuring 19.1 cm (Figure 1).

**Table 1:** Laboratory test results

LFT/Liver Synthetic Function Panel							
	Admission	Discharge	2 week follow-up	1 month follow-up	6 month follow-up	9 month follow-up	Range
AST	1405	1085	453	806	232	55	15 - 37 U/L
ALT	1074	737	500	1059	222	53	13 - 61 U/L
Alk Phos	291	240	389	447	243	90	45 - 117 U/L
Total Bilirubin	15.8	14.3	5.6	4.6	0.7	0.3	0.2 - 1 mg/dL
Direct Bilirubin	13.5	11.1	4.5	3.3	0.4	< 0.2	0.0 - 0.2 mg/dL
PT/INR	14.2 /1.23	14.1/1.1	-	13.5/1.0	13.0/1.0	-	9.7 - 13.0 sec / .83 - 1.09
Albumin	2.2	2.3	2.8	3.4	3.3	3.5	3.4 - 5.0 g/ dL
Viral Hepatitis Panel					Inflammatory Labs		
	Admission	Range				Admission	Range
Hepatitis A IgM Ab	Non-reactive	Non-reactive			ESR	81	0 - 10 mm/hr
Hepatitis A Ab Total	Reactive	Non-reactive			CRP	2.7	0.00 - 0.3 mg/dL
Hep Bs Ag	Non-reactive	Non-reactive					
Hep Bs Ab	Reactive	Non-reactive			Iron Panel		
Hep Bs Ab Index	> 1000.00	< 10 mIU/mL				Admission	Range
Hep B Core Total Ab	Reactive	Non-reactive			Iron	153	50 - 175 ug/dL
Hep B Core IgM Ab	Non-reactive	Non-reactive			TIBC	185	250 - 450 ug/dL
Hep C Ab Diagnostic	Non-reactive	Non-reactive			Iron Saturation	82	17.5 - 39 %
Cytomegalovirus Viral DNA Quant	98	< 31.2 IU/mL			Ferritin	4769	8 - 388 ng/mlL
Immune Serology Panel					Other Labs		
	Admission	Range				Admission	Range
Proteinase 3	<3.5	0.0 - 3.5 U/mL			Ceruloplamisn	48	20 - 60 mg/dL
c-ANCA Ab Titer	<1:20	Neg: <1:20			Serum Copper	180	69 - 132 ug/dL
p-ANCA Ab Titer	<1:20	Neg: <1:20					
Atypical p-ANCA Ab Titer	<1:20	Neg: <1:20					
ANA Ab Titer	1:160	Neg: <1:80					
IgG	3668	700 - 1600 mg/dL					
Anti-Nuclear Ab Pattern	Cytoplasmic	N/A					
Smooth Muscle & RNP Interpretation	108 U (Moderate to strong Positive)	0 - 19 U					
Smooth Muscle Ab Titer	1:320	Neg: <1:20					
Myeloperoxidase Ab	<9.0	0.0 - 9.0 U/mL					
Mitochondrial Ab Titer	<20.0	0.0 - 20.0 U					
Soluble Liver Ag IgG Ab	0.9	0.0 - 20.0 U					
Liver/Kidney Microsomes Ab Titer	<1:20	Neg: <1:20					

**Figure 2:**
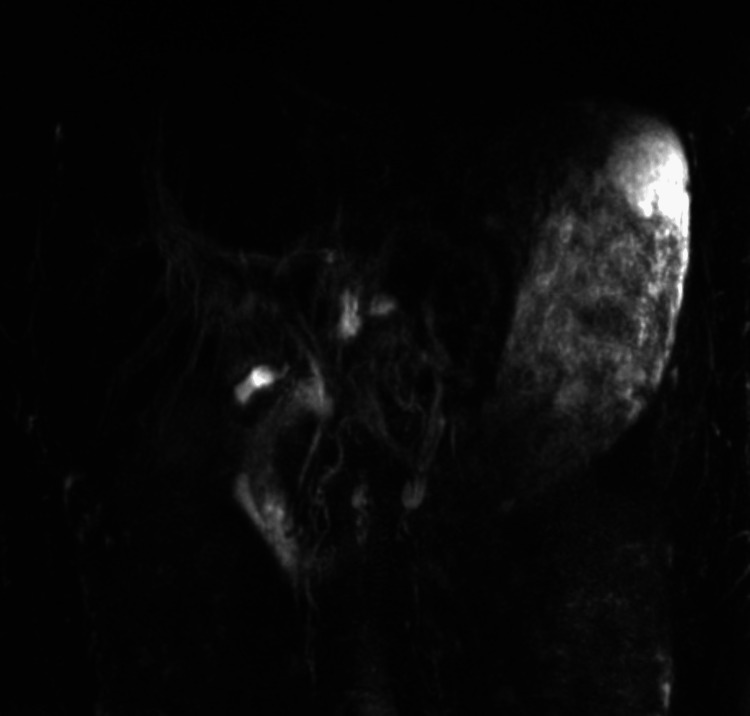
Magnetic resonance cholangiopancreatography overview

**Figure 3:**
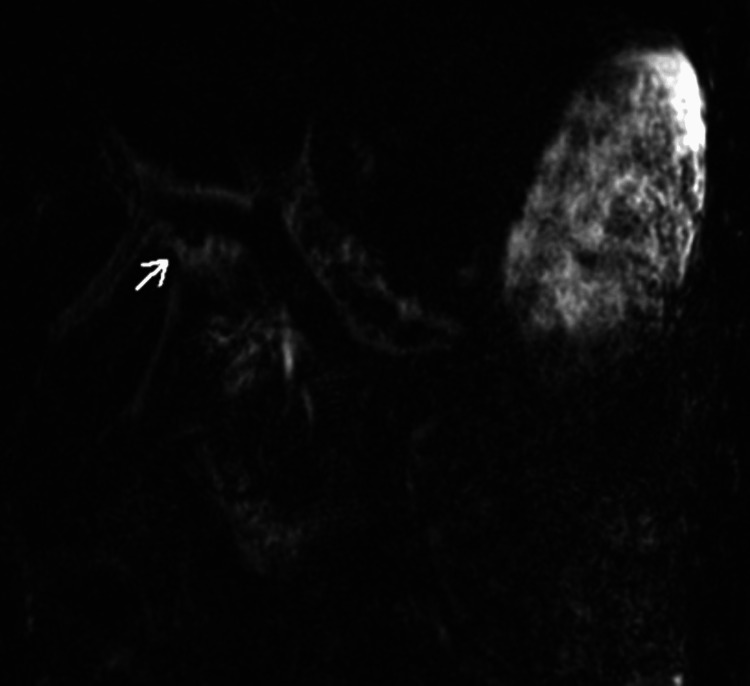
Magnetic resonance cholangiopancreatography demonstrating preiportal edema surrounding the portal veins

Magnetic resonance cholangiopancreatography did not identify biliary obstruction but did show mild hepatosplenomegaly, periportal fluid, without choledocolithiasis or pancreaticobiliary ductal dilatation (Figures 2, 3).

**Figure 4:**
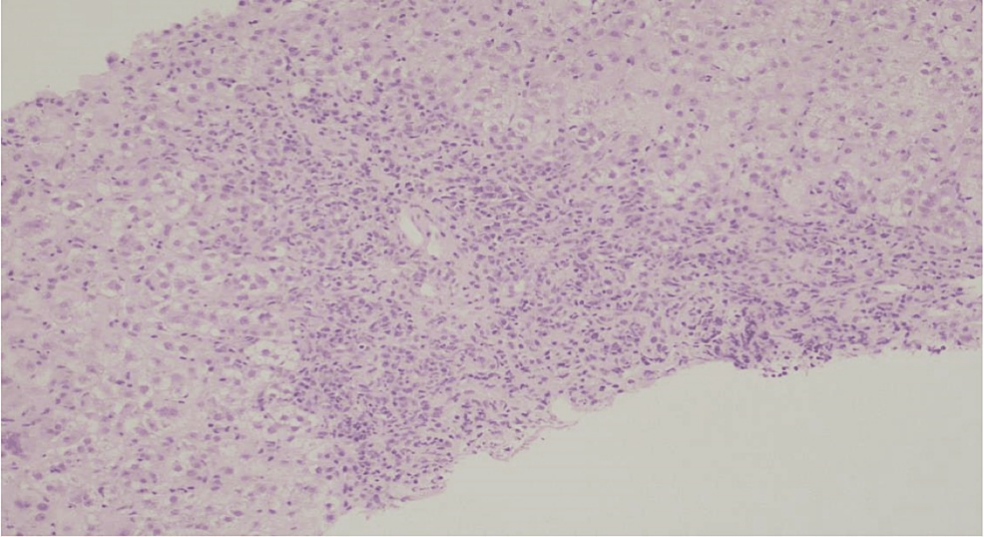
Liver biopsy under the microscope The portal tracts show marked expansion with mainly plasma cells, lymphocytes and few eosinophils. H&E, 100x.

**Figure 5:**
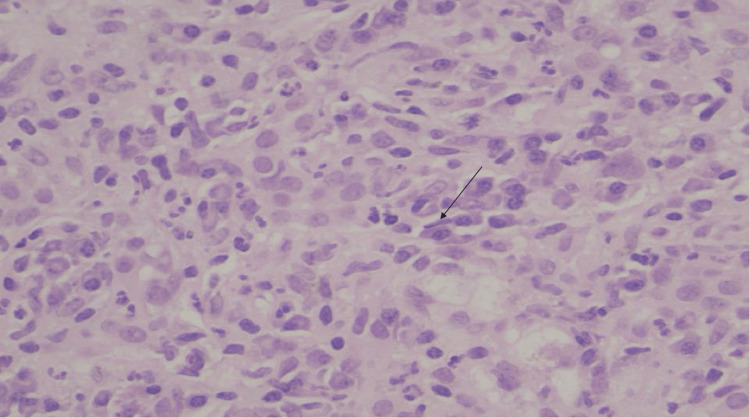
Liver biopsy under the microscope, zoomed in The portal tracts show plasma cells (highlighted by arrow) rich in the inflammatory infiltrate. H&E, 200x.

**Figure 6:**
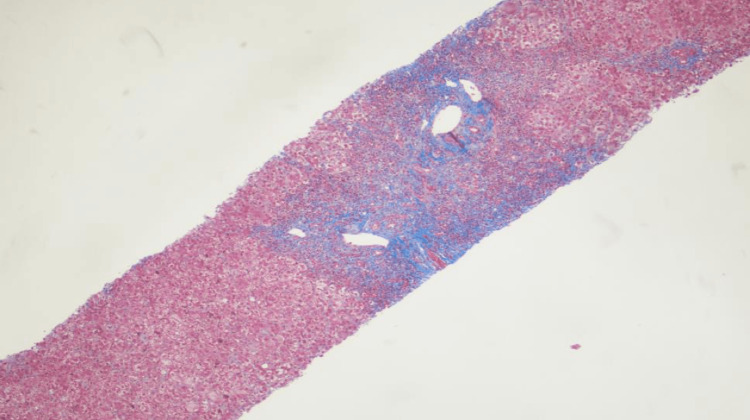
Liver biopsy under the microscope with trichrome stain Periportal fibrosis and mild perivenular fibrosis. H&E, 100x.

The patient was transferred to a tertiary care center for further diagnosis and management of acute severe hepatitis. Liver biopsy was performed. The portal tracts showed marked expansion by plasma cell-rich infiltrates with moderate ductular reaction (Figures 4, 5). The liver parenchyma revealed moderate perivenular necroinflammation, hepatocellular cholestasis with feathery degeneration and scattered apoptotic bodies. Multinucleated hepatocytes were also noted. Trichrome stain showed portal fibrosis that focally extended to periportal areas without bridging formation (Figure 6). These findings were consistent with immune mediated hepatitis with severe activity (grade 4 of 4) and periportal fibrosis (stage 2 of 4).

Given the patient’s presentation, positive anti-smooth muscle antibodies, positive ANA titers, elevated IgG antibodies, and the above liver biopsy findings the diagnosis of AIH was made. He was treated inpatient with a N-acetylcysteine drip and steroids. He was discharged on a 16-day course of prednisone taper, along with azathioprine. Additionally, he was prescribed valganciclovir on discharge given he had tested positive for CMV serology, which he did not take secondary to cost. At the time of discharge, liver function tests did not show significant improvement when compared to admission levels (Table 1).

At the initial follow-up visit two weeks after discharge from the hospital the patient had significant improvement of the aspartate aminotransferase (AST), alanine aminotransferase (ALT), and alkaline phosphatase (ALP). At subsequent follow-up approximately two weeks later, the patient had relapse of AIH after rapid steroid taper. Routine blood work at this visit showed an increasing ALT, AST, and alkaline phosphatase (Table 1). Prednisone was increased to 40 mg daily with no subsequent adequate improvement in liver function tests, and thus he was started on an eight-week steroid re-cycle with prednisone and azathioprine. He also started on Pneumocystis Jiroveci prophylaxis with atovaquone, and amlodipine for steroid-induced worsening of his hypertension.

## Discussion

The patient discussed has no high-risk behavior such as illicit drug use, alcohol use, or high-risk sexual behavior that would explain an elevation of transaminases. Although direct bilirubin was markedly elevated, MRCP did not show any intrinsic or extrinsic biliary tract obstruction or hepatic tumors. Hepatitis serology indicated evidence of previous hepatitis A and hepatitis B, but no active hepatitis was detected. Although iron saturation and ferritin were elevated, no iron deposits were detected in the hepatocytes or Kupffer cells in the liver biopsy. Additionally, his longstanding home antihypertensive, Preterax, which is a combination ACE inhibitor and thiazide-like diuretic, is not known to be associated with auto-immune hepatitis [6].

Positive CMV PCR does not argue against the diagnosis of SARS-CoV-2-induced AIH, as the patient did not receive appropriate treatment for CMV, still, his liver enzymes improved. Additionally, CMV staining on the liver biopsy was negative. The positive ANA, anti-smooth muscle antibodies, elevated IgG antibodies, and the specific histologic findings support the diagnosis of type-1 AIH. This, along with the clinical presentation and timing of symptoms in relation to SARS-CoV-2 infection, supports the diagnosis of SARS-CoV-2-induced AIH. 

This case demonstrates the presenting complaints, diagnostic findings, and therapy for our patient diagnosed with SARS-CoV-2-induced AIH and highlights these findings which are rarely seen after SARS-CoV-2 infection. There are many unique factors in this case. Firstly, SARS-CoV-2-induced AIH itself is a unique diagnosis, with less than a handful of documented cases, this being the third [2]. AIH itself has a low prevalence of 31.2/100,000 in the United States [3].

Another interesting detail about this particular patient is the sex. AIH is seen predominantly in females, with a 4:1 prevalence for Type I, and a 10:1 for Type II [7]. Finally, this patient developed AIH secondary to SARS-CoV-2 infection despite having been fully vaccinated against the SARS-CoV-2 virus months prior to infection. This may indicate that the COVID vaccine may not be protective against many post-COVID-19 complications, specifically autoimmune complications like AIH. The treatment course was complicated by relapse of AIH due to a rapid steroid taper. This highlights the possibility that in SARS-CoV-2-induced AIH patients may require a longer steroid taper to achieve disease remission.

## Conclusions

This case highlights a unique patient who developed AIH secondary to SARS-CoV-2 infection. It is important for physicians to consider AIH as a cause of abnormal liver function tests or even liver failure in the setting of active or recent SARS-CoV-2 infection. Missing the diagnosis or attributing hepatic function abnormality to a recent acute illness or medications without proper workup, might carry the risk for progressive or permanent liver damage requiring liver transplantation.
